# Knowledge about Anaesthesia and Anaesthesiologist Amongst General Population in India

**Published:** 2009-04

**Authors:** S K Mathur, S K Dube, Sunil Jain

**Affiliations:** 1 Reader, Department of Anaesthesiology, Institute of Medical Sciences, Banaras HinduUniversity, Varanasi-221005, INDIA; 2 P.G.Student, Department of Anaesthesiology, Institute of Medical Sciences, Banaras HinduUniversity, Varanasi-221005, INDIA; 3 Ex. M.D.Student, Department of Anaesthesiology, Institute of Medical Sciences, Banaras HinduUniversity, Varanasi-221005, INDIA

**Keywords:** Knowledge, General population, Perception, Anaesthesiology, Anaesthesiologist

## Abstract

**Summary:**

Anaesthesiologists are playing a decisive role in patient management. Present day anaesthesiology is based on the use of newer and safer drugs, better patient monitoring, pain management and critical care. But the general public knows little of these developments. The study was undertaken to assess the perception regarding the anaesthesiology and anaesthesiologists among the general population. The present study was conducted on 300 persons (patient, patient's attendant and medical undergraduates) between 18-75 years of age to assess the knowledge regarding anaesthesiology and the anaesthesiologists. All collected data were categorized into 5 groups as per the educational status of the study population. Perception of anaesthesiologist as a doctor in illiterate, graduate and postgraduate population was 19.51%, 58.57% and 87.88% respectively. Anaesthesiology as a separate medical discipline was not known to 100%, 73.87%, 64.29% and 51.52% of the illiterate, upto matriculation, graduate and postgraduate population respectively. Among the population who knew something about general anaesthesia, none from upto matriculation and 33.87%, 44.83% and 100% from the graduate, post graduate and medical undergraduate groups respectively knew that anaesthesia is administered with specialized equipments along with monitoring. Illiterates did not know about regional anaesthesia, while most of others had some knowledge about it. The results of the study reflect the wide spread ignorance and misconceptions about anaesthesiology and anaesthesiologists still prevalent in public in India.

## Introduction

Anaesthesiology today is an upcoming multimodality specialty in medical science with its spectrum ranging from perioperative patient care to pain management, critical care and palliative care. Anaesthesiologists today are playing a decisive role in patient management. As we talk of newer and safer drugs, better drug delivery systems and formulation of optimal management plans in terms of better perioperative management of vital functions and critical care we tend to ignore the fact that the general population understands little of these developments The problems of image and status of the anaesthesiologists in the eyes of the medical and lay communities are not new^1^. With the changing health care environment and advancement in anaesthesiology, the patients and general public needs to be educated^2^. The need of the time is to highlight anaesthesiology as a separate medical discipline in both audiovisual as well as print media. But before we suggest anything we actually need to know the level of awareness about anaesthesiology and the anaesthesiologist among the general population. The present study was conducted to assess general public's perception about anaesthesiologist and the practice of anaesthesiology.

## Methods

This crossectional study was focused on studying the awareness among the general population (patient, patient's attendant and medical undergraduates who are in their preclinical years) regarding anaesthesiology as a separate medical discipline, anaesthesiologist as a doctor and different techniques of anaesthesia. A pilot study was under taken in 15 individuals, to ensure that the questionnaire could easily be understood and if required any modification in the questionnaire could be made before proceeding for the study further. The study population was between 18-75 years of age. Persons who refused to participate, who were hard of hearing or those unable to answer questions due to poor medical condition were excluded from the study. The participants were explained that their participation in the study is totally voluntary and their responses will be kept confidential. The questionnaire had three parts. The first part of the questionnaire was about demographic information. The second part of the questionnaire was designed to assess the knowledge regarding the anaesthesiologist. The third part of the study was based on assessment regarding anaesthesiology and anaesthesia techniques. The questionnaire is given below.

## Questionnaire

### Part 1 Demographics

**Table:** 

Name	Occupation:
Age/Sex	Educational status:
	Illiterate
	Matriculation and below
	Graduation and below
	Post graduation and above
	Medical undergraduates

### Part 2 Knowledge regarding anaesthesiologist

**Table:** 

Do you have any knowledge regarding	
Anaesthesiologist as a Doctor?	YES/NO
If yes, then how?	
Hear say	
Told by the attending physician or surgeon	
Read somewhere	
From previous operation	
What is the role of Anaesthesiologist in your/patient's treatment?	
As a skilled assistant to surgeon	
Other definitive role in the operation theater	
No idea	
What an anaesthesiologist does in the operation theater?	
Only administers drug once and goes away	
Administers drug and monitors patient intra operatively	
Does something in the postoperative period as well	
What exactly an anesthesiologist does in the postoperative period?	
Monitors the patient only	
Does something for immediate post operative complication(s)	
Manages postoperative pain	
Who manages the post op. complications in the recovery room?	
Surgeon concerned	
Other physician	

### Part 3 Knowledge regarding anaesthesiology and anaesthetic techniques:

**Table:** 

Do you have any knowledge regarding anaesthesiology as a separate medical discipline?	YES/NO
What are the different kinds of anaesthesia techniques?	
General	
Regional	
No idea	

### Part 3(A) Knowledge regarding general anaesthesia

**Table:** 

Drugs used in general anaesthesia can be	
Only inhalational	
Only intravenous	
Can be both	
No idea	
Name some drugs used in present day general anesthesia	
Chloroform	
Ether	
Other Inhalational agents	
Intravenous agents	
No idea	
How general anaesthesia is given?
Using certain vapor in a handkerchief without monitoring
Using certain vapor in a handkerchief with monitoring
By certain specialized equipments without monitoring
By certain specialized equipments with monitoring	No idea
Do you have any knowledge regarding the complications related to general anesthesia?	YES/NO

### Part 3(B) Knowledge regarding regional anaesthesia

**Table:** 

Do you have any knowledge regarding the techniques of regional anesthesia?	YES / NO
If yes then do you have any idea about Local/Spinal/Epidural anaesthesia?	
Do you have any knowledge regarding complications related to regional anaesthesia?	YES / NO
Do you have any knowledge regarding the advantages of regional anaesthesia on other types of anaesthesia?	YES / NO

In part 2 of the questionnaire the answer “Read somewhere” included the knowledge acquired through text books, magazines, news papers etc. If the person answered that the anaesthesiologist has some definitive role in his/her/patient's treatment then he/she was asked “What does an anaesthesiologist do in the operation theater?” The knowledge regarding the role of the anaesthesiologist in the post-operative period among the persons who answered that the anaesthesiologist has some role in the post-operative period was analyzed by subsequent questioning. Those persons who answered that the anaesthesiologist has some role in the post-operative period but did not answer that the anaesthesiologist manages the immediate post-operative complication(s) were asked whether the immediate post-operative complications are managed by the surgeon concerned or there will be a physician other than the anaesthesiologist for it. In third part of the questionnaire those who answered general and/or regional anaesthesia as an anaesthetic technique were asked some specific questions related to general and/or regional anaesthesia. The persons who did not answer general/regional anaesthesia were not questioned further. If the person was able to name any of the inhalational agents other than chloroform/ether then the person was considered to have some knowledge about the other inhalational agents. All the data were categorized into five groups as per the educational qualification of the subjects.

## Results

Of the 300 persons 174 were male and 126 were female. Study population was divided into 5 groups on the basis of literacy level (Table 1).

**Table 1:** Demography (n=300)

Group	No of persons	Male	Female
1 Illiterate	41 (13.67%)	24 (58.54%)	17 (41.46%)
2 Matriculation and below	111 (37.00%)	61 (54.95%)	50 (45.05%)
3 Graduation and below	70 (23.33%)	40 (57.14%)	30 (42.86%)
4 Post graduation and above	33 (11.00%)	17 (51.52%)	16 (48.48%)
5 Medical undergraduates	45 (15.00%)	32 (71.11%)	13 (28.89%)
Total	300 (100%)	174 (58.00%)	126 (42.00%)

The results of the questions related to the knowledge regarding anaesthesiologist are shown in Table 2. Among the persons who answered that the anaesthesiologist does something in the post-operative period 86.21% in group 5 and 76% in group 3 believed that the anaesthesiologist only monitors the patient, but 58% in group 3 answered that the anaesthesiologist manages the immediate post-operative complication(s). The perception regarding the management of post operative complication(s) is summarized in Table 3. The results of the questions pertaining to the knowledge regarding anaesthesiology are shown in Table 4.

**Table 2:** Knowledge regarding anaesthesiologist

QUESTION	Group 1 (n= 41)	Group 2 (n= 111)	Group 3 (n= 70)	Group 4 (n= 33)	Group 5 (n= 45)
1. Anaesthesiologist is a doctor?					
• Yes	8(19.51%)	49(44.14%)	41(58.57%)	29(87.88%)	45(100%)
• No	33(80.49%)	62(55.86%)	29(41.43%)	4(12.12%)	0
If yes then how					
• Hear say	8(100%)	12 (24.49%)	4(9.76%)	8 (27.59%)	4(8.89%)
• Told by attending physician/surgeon	0	25(51.02%)	12(29.27%)	4 (13.79%)	0
• Read some where	0	12(24.49%)	21(51.22%)	4(13.79%)	41(91.11%)
From previous surgery	0	0	12(29.27%)	13 (44.83%)	0
2 Role of anaesthesiologist in treatment?					
• Skilled assistant to surgeon	33(80.49%)	41 (36.94%)	4(5.71%)	4 (12.12%)	0
• Other role in O.T.	8(19.51%)	70 (63.06%)	58 (82.86%)	29 (87.88%)	45(100%)
• No idea	0	0	8(11.43%)	0	0
3. Role in the O.T.					
• Administers drug once	8(100%)	46 (65.71%)	37 (63.78%)	4 (13.79%)	0
• Administers drug and monitors patient intra operatively	0	25(35.71%)	21(36.20%)	25 (86.21%)	45 (100%)
• Also works in post operative period	4 (50%)	41 (58.57%)	50 (86.20%)	29(100%)	29(64.44%)
4. Role in the post operative period?					
• Monitors patient only	4(100%)	41(100%)	38(76.00%)	4 (13.79%)	25(86.21%)
• Manages post operative complications	0	0	29 (58.00%)	0	0
• Manages post operative pain	0	0	8 (16.00%)	25 (86.21%)	4 (13.79%)
complications					
O.T.: Operation Theater					

**Table 3:** Knowledge regarding management of immediate postoperative complications

QUESTION	Group 1 (n= 4)	Group 2 (n=41)	Group 3 (n= 50)	Group 4 (n= 29)	Group 5 (n= 29)
Number of persons who did not	4(100%)	41(100%)	21(100%)	29(100%)	29(100%)
think that the immediate					
postoperative complication(s)					
are managed by anaesthesiologists					
If not anaesthesiologist then who else?					
• Surgeon concerned	4(100%)	16 (39.02%)	12(57.14%)	17 (58.62%)	0
• Other physician	0	25 (60.98%)	9 (42.86%)	12 (41.38%)	29 (100%)

**Table 4:** Knowledge regarding anaesthesiology

QUESTION	Group 1 (n= 41)	Group 2 (n=111)	Group 3 (n= 70)	Group 4 (n= 33)	Group 5 (n= 45)
1. Is anaesthesiology a separate medical discipline?					
• Yes	0	29 (26.13%)	25 (35.71%)	16 (48.49%)	45 (100%)
• No	41 (100%)	82 (73.87%)	45 (64.29%)	17 (51.52%)	0
2. Anaesthesia techniques?					
• General anaesthesia	12 (29.27%)	78 (70.27%)	62 (88.57%)	29 (87.88%)	45 (100%)
• Regional anaesthesia	0	41 (36.94%)	49 (70.00%)	16 (48.49%)	45 (100%)
• No idea	29 (70.73%)	29 (26.13%)	4(5.71%)	4 (12.12%)	0

Results of the questions related to general anaesthesia are shown in Table 5. On being questioned, the persons in each group who answered general anaesthesia as an anaesthesia technique, about the drugs used in general anaesthesia, 100% people in group 1 and 74.36% people in group 2 told the drugs to be only inhalational agents. In group 4, 3 and 2, 86.21%, 59.68% and 46.15% respectively named chloroform as a drug used in present day general anaesthesia. Sixty four percent of people in group 5 carried the impression that ether is used in present day general anaesthesia as compared to 31% people in group 4. In group 5 only 17.78% people named some intravenous and other inhalational agents. Interestingly, large majority of population in group 1 (100%), group 2 (84.62%) and group 3 (72.58%) still believe that the general anaesthesia is given by using certain vapors in a handkerchief without monitoring. One interesting finding is that more people in group 4 knew about the complications of general anaesthesia than those in group 5. Table 6 shows the results of the questions related to regional anaesthesia.

**Table 5:** Knowledge regarding general anaesthesia

QUESTION	Group 1 (n= 12)	Group 2 (n= 78)	Group 3 (n= 62)	Group 4 (n= 29)	Group 5 (n= 45)
1. Agents used in G.A. can be?					
• Only inhalational	12 (100%)	58 (74.36%)	12 (19.36%)	12 (41.38%)	0
• Only intravenous	0	0	9(14.51%)	5 (17.24%)	0
• Can be both	0	20 (25.64%)	41(66.13%)	12 (41.38%)	45(100%)
• No idea	0	0	0	0	0
2. Name drugs used in present day general anaesthesia					
• Chloroform	0	36 (46.15%)	37 (59.68%)	25 (86.21%)	45(100%)
• Ether	0	8 (10.26%)	8(12.90%)	9 (31.03%)	29 (64.44%)
• Other Inhalational agents	0	0	0	0	8 (17.78%)
I.V. Agents	0	0	0	0	8 (17.78%)
• No idea	12(100%)	42 (53.85%)	25 (40.32%)	4(13.79%)	0
3. How general anaesthesia is given?					
• Using handkerchief without monitoring	12 (100%)	66 (84.62%)	45 (72.58%)	4(13.79%)	0
• Using handkerchief with monitoring	0	0	0	0	0
• By specialized equipments without monitoring	0	8 (10.26%)	8 (12.90%)	12 (41.38%)	0
• By specialized equipments with monitoring	0	0	21(33.87%)	13 (44.83%)	45(100%)
• No idea	0	4(5.13%)	13(20.97%)	0	0
4. Any idea regarding the complications of general anaesthesia?					
• YES	0	4(5.13%)	8 (12.90%)	12 (41.38%)	16 (35.56%)
• No	12 (100%)	74 (94.87%)	54(87.10%)	17 (58.62%)	29 (64.44%)
G.A.: General Anaesthesia					

**Table 6:** Knowledge regarding regional anaesthesia

QUESTION	Group 1 (n= 4)	Group 2 (n=41)	Group 3 (n= 50)	Group 4 (n= 29)	Group 5 (n= 29)
1. Knows regional anaesthesia?	N.A.				
• Yes	N.A.	37(90.24%)	49(100%)	16(100%)	45(100%)
• No	N.A.	4(9.76%)	0	0	0
If yes then idea regarding	N.A.				
• Local anaesthesia	N.A.	25(67.56%)	41(83.67%)	12(75%)	45(100%)
• Spinal anaesthesia	N.A.	16(43.24%)	21(48.97%)	12(75%)	37(82.22%)
• Epidural anaesthesia	N.A.	0	0	0	0
2. Knows complications of R.A.?	N.A.				
• Yes	N.A.	0	0	0	20(44.44%)
• No	N.A.	41(100%)	49(100%)	16(100%)	25(55.56%)
3. Knows advantage of R.A.?	N.A.				
• Yes	N.A.	0	0	0	0
• No	N.A.	41(100%)	49(100%)	16(100%)	45(100%)
N.A.: Not Applicable; R.A.: Regional Anaesthesia					

## Discussion

The problems of image and status of the anaesthesiologists in the eyes of the medical and lay communities are not new.^1^ Regarding issues relating to the status and image of the specialty many, if not all, practicing anaesthesiologists have struggled at some point. Development of anaesthesia as a specialty has enabled the advancements in surgical management and critical care. In our study a large portion of population with below graduation level education did not even know the anaesthesiologists as a doctor. In previous studies conducted elsewhere 50% to 88.7% people knew the anaesthesiologist as a doctor.^2^–^5^

In our study the source of information regarding the anaesthesiologist as a doctor was by virtue of reading some where or from the attending physician/surgeon in majority of the population. The electronic and print media has a tremendous potential to educate the general population, but this potential has always being under-utilized. If the patients have beforehand knowledge through audiovisual or print media about anaesthesiology then they may have an option to enquire and choose their anaesthesiologist so that a less qualified or unqualified person won't be involved in the practice of anaesthesiology.

Educating the physicians or surgeons regarding our discipline may improve the knowledge that the patients get from them regarding our role in patient management. A survey of 2500 pediatricians who were either involved in preoperative examination of the children or of the opinion that they should routinely examine children preoperatively, revealed that the knowledge of relevant anaesthetic issues was lacking in them.^6^

We found that majority of the illiterate people knew the anaesthesiologist as a skilled assistant to surgeon. But the population with education level of matriculation and above had an impression that the anaesthesiologists have some definitive role in the operation theatre. Upon asking about the role of the anaesthesiologists in the operation theatre most of the people answered that the anaesthesiologists administers drug once and goes away. This was in contrast to the findings of the surveys conducted in developed countries where a majority of patients felt that the anaesthesiologist stays during operation to look after their vitals.^4^,^7^ Role of the anaesthesiologists after induction was not clear to many patients in previous studies.^8^,^9^

As on today, much of the emphasis in anaesthesiology is on intra-operative patient monitoring to improve patients' safety. But in our study majority of the population was unaware of intra-operative patient's monitoring which was similar to the finding of the study by Shevde and Panagopoulos.^8^ Regarding the role of the anaesthesiologist in the peri-operative patient care the general population needs to be educated that the anaesthesiologist is in fact an internist in the operating room. In our study we found that majority of the population did not have any idea regarding anaesthesiology as a separate medical discipline. This was in contrast to the study finding of Gurunathan and Jacob.^9^

Most of the people in our study knew general anaesthesia as an anaesthesia technique. Upon asking about general anaesthesia most of the people knew the drugs used in general anaesthesia as inhalational agents and the mode of administration is through a handker-chief without monitoring. Excepting the medical undergraduates none knew about the intravenous and other inhalational anaesthetic agents. Present day anaesthesiology is based on use of safer drugs and drug delivery systems. In our study majority of the population was ignorant about these developments (Table 5). Most of the people knew about the techniques of regional anaesthesia with majority answering local anaesthesia as a type of regional anaesthesia. Now days, the advancements in regional anaesthesia have allowed many complex surgical procedures to be performed under regional anaesthesia. But in our study no one knew about advantages of regional anaesthesia on other types of anaesthesia.

The finding that is more disappointing in our study is the lack of awareness regarding our discipline among the medical undergraduates. The role of anaesthesiologist in the post-operative period and pain management is unclear to many medical undergraduates (Table 2 and 3). Many of the medical undergraduates were ignorant about regional anaesthesia, the drugs used as well as the complications of general anaesthesia (Table 5 and 6). This lack of awareness may be one of the reasons why the medical undergraduates are not eager to pursue a carrier in anaesthesiology. The need to reform the existing medical curriculum in terms of increasing the hours of anaesthesiology teaching and compulsory internship posting in anaesthesiology should be emphasized so that undergraduate students can have better exposure to different domains of anaesthesiology.

The patients remember more about their surgeons than their anaesthetist^10^,^11^ may be because of the limited time we spend in communicating with patients resulting in not obtaining adequate patient satisfaction as compared to other specialists. The education of other health care professionals may be enhanced by publishing papers in their journals and by participating in multidisciplinary hospital committees.^1^ Information that increases public awareness of the role of anaesthesiologist will contribute towards improving the image of anaesthesia.^5^ To improve our image in the community the needed efforts can be directed towards improving our communication with patients and increasing our exposure in the community via newspapers, audiovisual media and lectures.

Our search for anaesthesiology related articles in three major local news papers which publish health related topics on weekly basis revealed that the topics related to anaesthesiology were outnumbered by articles related to other specialties. The American Society of Anaesthesiologists sponsors an annual media award to recognize outstanding contributions in the patients' education. A task force on public education and information by the American Society of Anaesthesiologists recommended that public education program should take place at the grass roots level and has appointed a manager of state programs to facilitate this endeavor.^2^ Similar initiative if taken in our country may strengthen our endeavor to improve our image among the general population.

Better knowledge about various anaesthesia techniques and their possible complications in various conditions of patients may reduce the number of medico legal litigations. A good communication with the physician as judged by the patient is associated with lower incidence of malpractice litigation.^12^ Well informed patients can select their anaesthesiologists which can help in improving the peri-operative care which in turn will reduce the morbidity. The study population in our study (which represents a section of population of northern India) and the small sample size are the few limitations to our survey. But the result of the study reflects the wide spread ignorance and misconceptions about anaesthesiology and anaesthesiologists still prevalent in public mind in India.

Thus we conclude that ignorance regarding the anaesthesiologists and anaesthesiology is still prevalent among the general population. To disseminate information about anaesthesia, the existing educational methods are to be evaluated and newer initiatives are to be looked for. The involvement of electronic and print media in educating general population, irrespective of their educational status, can have a strong impact on our effort in educating them. Our success in educating the public, other health care professionals and politicians about our role in patient management may play an important role in our future progress.
